# Proteome evaluation of human cystic echinococcosis sera using two dimensional gel electrophoresis 

**Published:** 2018

**Authors:** Fatemeh Sadat Sadjjadi, Mostafa Rezaie-Tavirani, Nayeb Ali Ahmadi, Seyed Mahmoud Sadjjadi, Hakimeh Zali

**Affiliations:** 1 *Proteomics Research Center, Faculty of Paramedical Sciences, Shahid Beheshti University of Medical Sciences, Tehran, Iran*; 2 *Department of Parasitology and Mycology, School of Medicine, Shiraz University of Medical Sciences, Shiraz, Iran.*; 3 *School of Advanced Technologies in Medicine, Shahid Beheshti University of Medical. Sciences, Tehran, Iran*

**Keywords:** Proteomics, cystic echinococcosis, Cluster, Hydatid cyst, 2-D electrophoresis (2‐DE)

## Abstract

**Aim:**

Detection of protein expression changes in human cystic echinococcosis sera by 2D gel electrophoresis.

**Background:**

Diagnosis and successful treatment of cystic echinococcosis (CE) is a major challenge, up to now. Identification of related expressed proteins using proteomics tools and bioinformatics analysis of patients’ sera have not been investigated, so far.

**Methods:**

Sera from eight confirmed CE patients and three healthy controls were collected, tested by 2-DE for total protein separation of serum and analyzed using proteomics and bioinformatics methods. The gels were stained by Coomassie blue followed by scan imaging of the gels. The protein spots in each gel were analyzed using progenesis same spots software. Proteins names were obtained from TagIdent server.

**Results:**

A total of 263 protein spots with different expression were detected in both normal and diseased samples. Comparison between diseased and normal gels showed the expression of 45 up-regulated protein spots with fold≥2 in diseased gel of which 10 were new proteins with statistical difference by normal gel (p-value<0.05). On the other hand, the expression of 50 down-regulated protein spots were observed of which 11 proteins have been suppressed. Clustering of all detected sera proteins (263) using correlation analysis, divided the proteins into 2 clusters based on up-regulated and down-regulated expression of proteins. Clustering results were approved by principal component analysis (PCA).

**Conclusion:**

Significant protein expression changes in human CE sera which is demonstrable by application of proteomics and bioinformatics analysis makes it an impressing tool for diagnosis of CE patients.

## Introduction

 Cystic echinococcosis (CE)/hydatidosis is one of the most serious parasitic diseases in human and animals around the world including the Middle East (1). It is caused by the larval stage of *Echinococcus granulosus* (*E. granulosus*), a zoonotic Platyhelminthes. Human can be infected accidentally by ingesting the contaminated water or vegetables with *E. granulosus* eggs. The highest rate of infection is in the Mediterranean region that Iran is a country in this area. The disease has a significant impact on human and animal health and cause social and economic problems in endemic areas (2). The annual surgical cases of human CE in Iran are reported to be 1–2 per 100,000 people (3). Diagnosis and treatment of CE is a most challenge, up to now (4). CE in humans is asymptomatic in the early stages, but over time some symptoms in patient have been appeared (5). The diagnosis of CE is based on serological methods including ELISA, CCIEP and imaging methods such as radiography, ultrasound sonography (US), CT, MRI (6,7) However, when the cysts are newly formed, radiology and ultrasound methods are not satisfactory (8). Serologic diagnosis is difficult due to cross-reaction with other diseases. In this regard, different antigens including B antigen in its native and recombinant forms has been used for serological diagnosis of human CE (9). But investigation of serum proteome in the view of proteomics could be desirable. Rapid detection leading to biomarkers serves as strong tool to prevent and control the progression of infectious diseases (10). Investigation of specific antibodies or antigens in human sera is of interest for diagnosis of CE patients. Reliable methods and protocols are required for accurate diagnosis of human CE (11). A novel approach has been employed for identification of candidate antigens for specific immunodiagnosis of human CE. The employed strategy has taken the advantage of the fact that a database of *E. granulosus* genome is now available which allows an immunoproteomics approach to be undertaken using sera from human CE patients (12). On the other hand, identification of expressed proteins using protein function prediction methods help the researchers to assign biological or biochemical roles of proteins. One of the most frequently used techniques for characterization of the protein components of biological materials is two-dimensional gel electrophoresis (2-DE). It is used to compare changes in protein level, modification, and degradation between case and control or treated and untreated samples (13). Proteomics changes can be revealed by gel image analysis after visualization by staining and identification of protein species using cluster analysis of proteins (14). In recent years, a few studies have been conducted on the proteome of CE fluid, protoscoleces, and adult stage of worm and most of them introducing proteins. Identification of proteins with high or less expression in CE patients’ serum, may introduce new candidates protein for serological diagnostics, drug and vaccine candidates (15-18). On the other hand, detailed proteomics analysis can lead to the identification of biomarkers for diagnosis or detection of CE in early stages. One of the main applied methods in proteomics study for separation of proteins is two dimensional gel electrophoresis (2DE). The information which is obtained from 2DE is huge, so application of this method which has been used for a number of infectious diseases (19-21), makes it an impressing tool for diagnosis of CE patients. On the other hand, new analysis methods for data mining is necessary for such diagnosis (22). Using strong software and servers for identification of new patterns in the data set could help the researchers in time and results. Clustering, principle component analysis (PCA) and correlation analysis could be performed with software. In the clustering, proteins are clustered into different groups with co-expression so that in each cluster the biological correlation could be detected (23). The present study used proteomics techniques followed by gel analysis bioinformatics methods on CE patients’ sera and healthy controls in the proteome level to explain the differences and similarities in sera of this important zoonotic disease in human. 

## Methods

**Sample collection**

The sera used in this study were obtained from Iranian CE patients as well as healthy controls after having specified informed consents. All the subjects were new cases, with no pregnancy, without taking medication, lack of other disease, and cysts in the liver.

This study was approved by the Ethics Committee of Shahid Beheshti University of Medical Sciences (ethical code: IR.SBMU.RETECH.REC.1396.537). A total of eight sera samples from CE patients were collected from CE patients who were undergone surgery for removal of their cyst and confirmed by pathology. A total three sera samples from healthy persons were used as control ones. CE patients and control sera have been frozen until use.

**Protein separation (2D gel electrophoresis)**

The frozen sera were defreezed in room temperature while they were vortexed on ice surface. All eight diseased samples and control sera were pooled separately and transferred into another micro-tubes and vortexed again. Protein concentration determined using 2D Quant kit (GE Healthcare). The first dimension of 2D electrophoresis was performed using 11 cm immobilized pH gradient (IPG) strip (pH 3-10). Before separation IPGs should be rehydrated. For rehydration 18 μl of sample was added to 180 μl of rehydration buffer consist of 7M urea, 2M thiourea, 2%CHAPS, 0.5 % ampholite, 40 mM DTT and 0.002% bromphenol blue. The protein mixture and rehydration buffer were loaded to IPG strip for 20 minutes. After rehydration, the first dimension has been performed with several voltage and time as (150v, 1hrs), (300v, 3hrs), (1000v, 1hrs), (6000v gradient, 2hrs) and (6000v, 2.5hrs). In the first dimension, the proteins were separated based on their isoelectric point. Next, the gels were equilibrated twice in equilibration buffers: (urea 7.2 gr, SDS 0.4 gr, Tris pH 8.8 1 ml, glycerol 3.428 ml) once by DTT 0.06 gr and once by IA 0.75 gr) in room temperature for 15 minutes. The second dimension of 2D electrophoresis was done using 2D HPE Double gel 12.5% (SERVA cat No.43862.00). IPG strips were transferred to the second dimension of gel. The gel was transferred into Flat Top tower device to separate the proteins based on their molecular weight. Then electric current was connected for four steps including: (100V, 7 mA, 1W, 30 min), (200V, 13mA, 3W, 30min), (300V, 20mA, 5W, 10min) and (1000V, 40mA, 25W, 2hrs) in 15° C temperature.

**Gel staining and gel de-staining**

Following electrophoresis, the gels were soaked in the Coomassie blue staining solution including Coomassie Brilliant Blue R250 0.3 gr, 85% orthophosphoric acid 18 ml and acetic acid 90 ml in 40° C temperature for 2 hr. Next, the gels were either destained overnight in destaining solution including 10% acetic acid while shaking.

**Figure 1:**
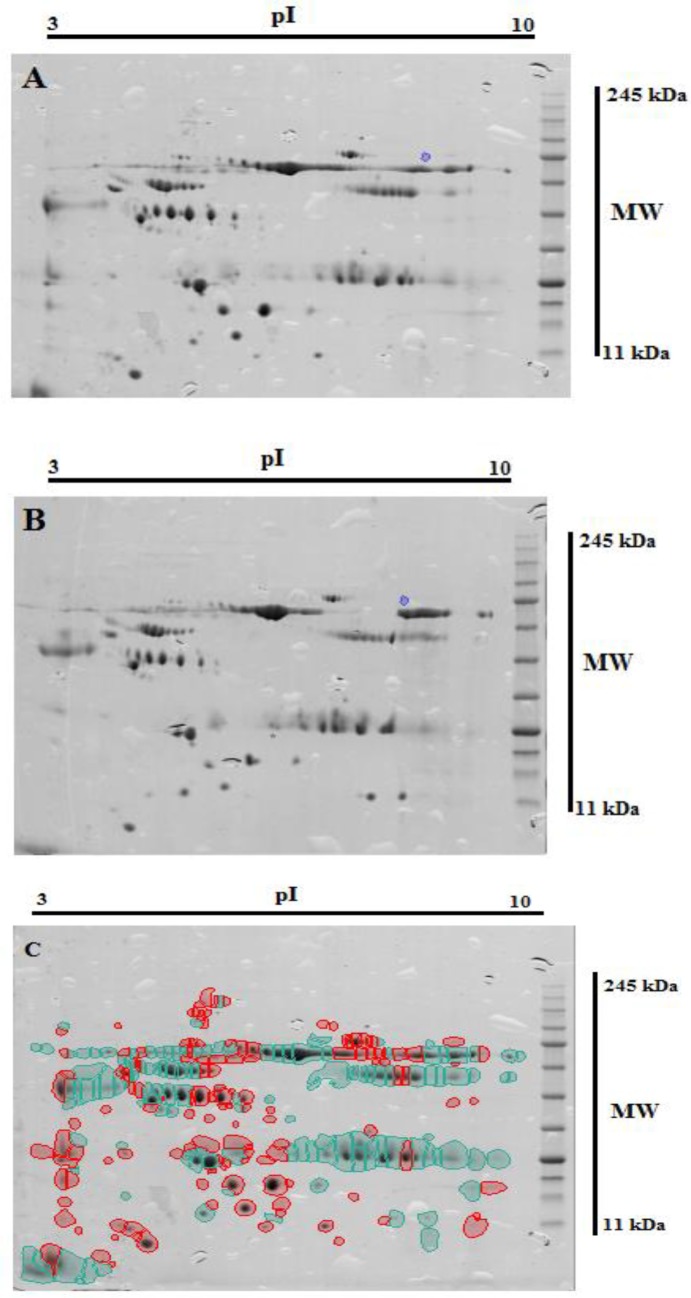
Proteins were separated on a pH 3-10 IPG strips in the first dimension and on 2D HPE Double gel 12.5% in second dimension 2D gel electrophoresis image scan of normal sample (A) and disease sample (B). Blue spots indicate up-regulated and red spots indicate down-regulated proteins in CE relative to healthy people (C

**Bioinformatics analysis**

2DE gels were scanned by Densitometer GS-800 (BioRad). The gels were analyzed using Progenesis same spot software. The software compares the processed gels, detecting protein spots, measures the color intensity of spots, spots alignment and performs statistical analysis. Statistical significance changes in proteins expression achieved by analysis of variance ANOVA (P-value <0.05) and fold change (fold>=2). Hierarchical clustering was used for comparing two groups and principal component analysis (PCA) was done for clustering confirmation. Significant spots identified using TagIdent tool (http://web.expasy.org/tagident/). 

## Results

2-DE was used for separating sera proteins in normal and disease samples. A total of 263 spots with different expression were identified in normal and disease samples by analysis of gels using Progenesis same spot software. The Comparison of protein spots between normal and CE patients showed that 134 and 129 spots had high and low expression in disease respectively (figure 1).

**Figure 2:**
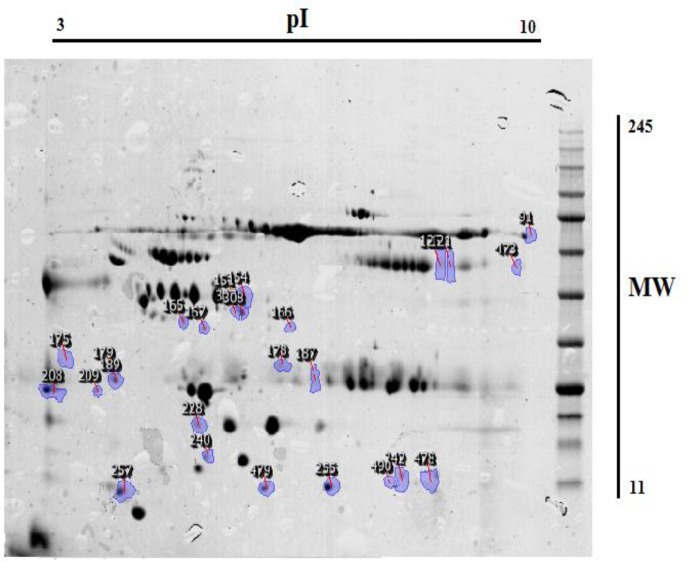
Protein spots number that analyzed showed in the gel

**Figure 3:**
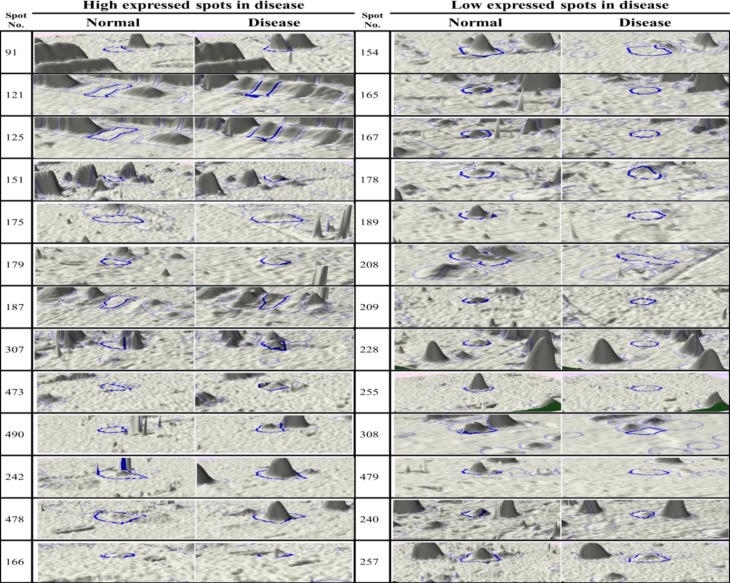
Three dimensional images of significant protein spots with different expression in two groups. New spots or suppressed spots can be seen in image clearly

**Table 1:** The protein predicted with TagIdent server based on their pI and MW. The last column shows that these proteins are up-regulated, down-regulated, new or suppressed in disease

**Spot No.**	**Protein description**	**Uniprot ID**	**pI**	**MW**	
91	Isoform 5 of Niban-like protein 2	Q86XR2-5	9.85	66253	New
121	E3 ubiquitin-protein ligase NEURL1B	A8MQ27	8.76	59270	New
125	5' exonuclease Apollo	Q9H816	8.63	60002	New
151	Target of EGR1 protein 1	Q96GM8-2	6.04	47890	New
175	Isoform 2 of RNA-binding protein 33	Q96EV2-2	3.85	30238	New
179	DFF35 of DNA fragmentation factor subunit alpha	O00273-2	4.48	29411	New
187	Isoform 5 of ADAMTS-like protein 1	Q8N6G6-5	7.09	27469	New
307	Isoform 3 of Hsp70-binding protein 1	Q9NZL4-3	6.05	44474	New
473	Isoform 2 of Uncharacterized protein KIAA0895	Q8NCT3-2	9.68	58304	New
490	Uncharacterized protein GAS8-AS1	O95177	8.05	12619	New
242	Keratin-associated protein 5-11	Q6L8G4	8.16	14610	Up
478	Isoform 2 of Acyl-coenzyme A thioesterase 13	Q9NPJ3-2	8.65	12367	Up
166	CX3C chemokine receptor 1	P49238	6.74	40397	Up
154	Receptor-type tyrosine-protein phosphatase	Q16827-3	6.15	47164	Suppress
165	Beta-actin-like protein 2	Q562R1	5.39	42003	Suppress
167	HLA class I histocompatibility antigen, A-11 alpha chain	P13746	5.66	38413	Suppress
178	Hematopoietically-expressed homeobox protein HHEX.	Q03014	6.71	30022	Suppress
189	Calcipressin-3	Q9UKA8	4.54	27492	Suppress
208	Acidic leucine-rich nuclear phosphoprotein 3	Q9BTT0-3	3.66	25125	Suppress
209	Uncharacterized protein C6orf106	Q9H6K1-2	4.3	25673	Suppress
228	Interferon alpha-4	P05014	5.58	19379	Suppress
255	ATPase inhibitor, mitochondrial	Q9UII2	7.22	9517	Suppress
308	LIM homeobox transcription factor 1-beta	O60663	6.22	44917	Suppress
479	Isoform 3 of Emopamil-binding protein-like	Q9BY08-3	6.38	10873	Suppress
240	Uncharacterized protein C3orf14	Q9HBI5	5.67	15007	Down
257	Protein S100-B	P04271	4.57	10582	Down

**Figure 4:**
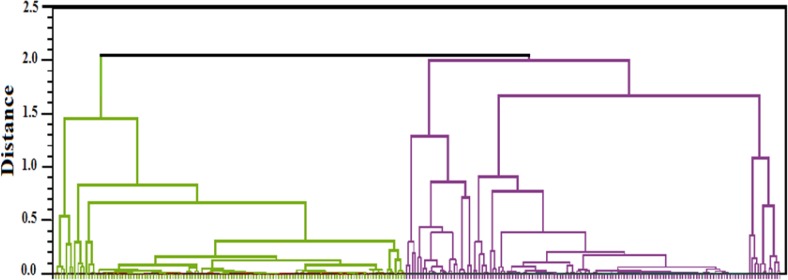
Hierarchical clustering of protein spots. The diagram indicates the cluster of 134 protein spots found up-regulated (left branches, in green) and 129 protein spots found down-regulated (right branch, in purple) in CE. All of the proteins with different expression have been classified into two groups: either increasing expression or decreasing the expression. These two groups are represented by two clusters

**Figure 5:**
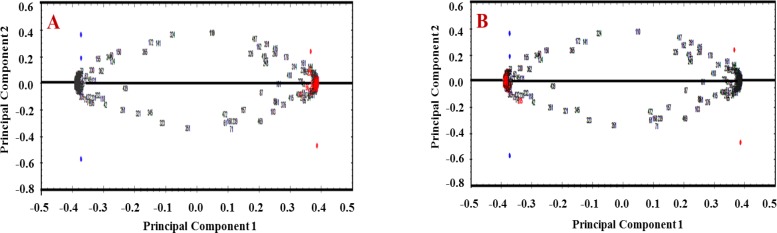
The PCA platform of normal and CE group. A) The PCA of 45 spot proteins was up–regulated in human CE (red spots), B) The PCA of 50 spot proteins were down-regulated in human CE (red spots). PCA confirmed clustering and there is no outlier in protein data set of both groups

Among these spots 95 proteins have significantly differences in both groups with p-value<0.05 and fold>=2 which 45 proteins have been high expressed in patient while 50 proteins had low expression. Also 10 new proteins have been identified and 11 proteins have been suppressed in patient sera are shown in figure 2. With more analysis significant spots of each group have been identified using TagIdent tool and the results are described in Table 1. The 3D images of these spots have been demonstrated in (figure 3). Differences between spot could be seen better in 3D shape. New spots, suppressed spots, up-regulated and down-regulated spots could be comprised clearly in 3D image. 

Hierarchical clustering of all detected sera proteins (263) using correlation analysis divided the proteins into 2 subgroups based on up-regulated expression of proteins and down-regulated expression of proteins (figure 4). The protein expression pattern in each cluster was resembled. So, the proteins of each cluster may have similar function or common signaling pathways. To confirm the clustering of expressed proteins using Principal Component Analysis on both normal and disease groups of samples. The PCA platform of normal and diseased groups have been shown in figure 5.

## Discussion

Immunoproteomics approach for diagnosis of human infectious diseases are a new and impressing tool (10, 24, 25). The present study which investigates proteomics and cluster analysis of human cystic echinococcosis sera using two dimensional gel electrophoresis, proteins name was predicted by TagIdent server other than experimental method. Due to the applied method, comparing to the results of experimental method is not possible in such studies (26). Proteomics tools and bioinformatics analysis of patients’ sera on protein expression levels related to the disease could help the scientists for more reliable diagnosis and follow up of the disease (27). In this regards, we performed proteomics analysis of sera from confirmed CE patients by 2DE for proteins which separated 263 proteins in the serum of CE patients. So, CE causes changes in protein expression in patients’ sera which could be used as a suitable method for antibody/antigen detection for diagnosis or follow up of CE patients. This has been already shown in pancreatic carcinoma, diabetes, gastric cancer and etc. (28-30). Diagnosis of the released molecules into patient’s sera especially in infectious diseases could be used as diagnostic or treatment targets (31). So, analysis of sera in CE patients and comparing it with normal people sera may help to a better understanding of the host–parasite relationship. Our gel analysis demonstrated 263 spots of which 45 spots were up-regulated and 50 spots were down-regulated significantly in patient sera. This shows that CE can change protein expression level in patients’ sera which could be a criteria for identification of infected people. Similar finding has been reported leptospirosis serum patients (19). Recent advances in 2DE and bioinformatics have provided significant tools for high-throughput data analyzing in proteomics approaches (32). Application of 2DE method for separation of proteins in the serum of CE patients and healthy people followed by bioinformatics analysis as has also been used for several infectious diseases such as schistosomiasis, hepatitis C and etc. (20, 21). Present study showed that 10 new proteins in CE patients. On the other hand, the expression of 11 proteins was suppressed in patients’ sera. Chemale and his colleagues also used 2DE for proteomics study on larval stage of *E. granulosus* and showed 15 prominent protein spots which shows that 2DE can be used for study of several aspect of *E. granulosus* (33). Also using 2DE combine MALDI-TOF/TOF on excretory/secretory products (ES) and antigenic proteins of *E. granulosus* adult worms has been led to identify 6 antigenic proteins (34). In our study, TagIdent server was used to identify proteins that changed their expression. TagIdent is a server that used for identification purposes in proteins by specification of their pI and Mw as estimated from the 2-D gel followed by specification of their error margins that reflect the known accuracy of these estimates. This method has been successfully used by Wilkins *et al*. (35). Among the expressed proteins, some maybe belong to parasite that has been secreted to patient’s sera and can be considered as antigenic proteins. To determine molecular mechanism of disease, protein identification with experimental methods such as mass spectrometry or bottom-up methods is necessary (36). Separated proteins have been analyzed with Progenesis same spot software which is a powerful tool for protein clustering and correlation analysis in many groups. Using correlation analysis divided the protein spots into 2 clusters based on up-regulated and down-regulated expression of proteins such that the protein expression pattern in each cluster was similar (37). This part is similar to the works on Malignant Gliomas in human which the authors reported that each cluster may have resembled and similar function or common signaling pathways (38). Principal component analysis (PCA) is a statistical technique that used to emphasize variation of data. This method often used to probe and visualize data easily. As has been shown in the result section, principal component analysis (PCA), which is usually used for outlier data and confirm clustering was applied to our data analysis and showed that there is no outlier in protein data set of both groups. Proteomics and metabolomics investigations used PCA to classify their data set that have been already used for metabolomics and proteomic data analysis of *Staphylococcus aureus* to classify its proteins and metabolites that responses to prolong stress (39).

In conclusion, using proteomics methods and bioinformatics analysis on sera of human CE patients comparing to healthy controls, the protein expression has been significantly shown to change in human CE sera and such alterations in serum protein expression levels which occurs in CE sera is demonstrable by bioinformatics analysis. These proteins will provide a better understanding of the disease and will help to predict new drug targets, new diagnostic biomarker and control strategies; although more researches are necessary.
